# Deterioration of filtering bleb morphology and function after phacoemulsification

**DOI:** 10.1186/1471-2415-13-17

**Published:** 2013-04-23

**Authors:** Monika Sałaga-Pylak, Małgorzata Kowal, Tomasz Żarnowski

**Affiliations:** 1 Chair of Ophthalmology, Medical University of Lublin, Chmielna Str No 1, Lublin, 20-079, Poland

**Keywords:** Intraocular pressure, Trabeculectomy, Phacoemulsification, Filtering bleb

## Abstract

**Background:**

Trabeculectomy remains the most efficient method of lowering he IOP applied for the treatment of glaucoma refractory to pharmacological treatment. Cataract is concerned as the most frequent late complication of trabeculectomy. The aim of the study was to analyse the effect of phacoemulsification with posterior chamber lens implantation on the morphology and function of filtering bleb in patients after previous successful trabeculectomy.

**Methods:**

The retrospective study included 122 eyes treated for primary open angle glaucoma, 50 eyes (study group) in which, after a successful trabeculectomy with5-Fluorouracil, phacoemulsification with posterior chamber lens implantation was performed, and 72 eyes (control group), in which only a successful trabeculectomy was conducted. The surgical success of the trabeculectomy was expressed as IOP < 17 mmHg.

**Results:**

In the group of patients subjected to both trabeculectomy and phacoemulsification, mean IOP was significantly higher than in the group of patients who underwent trabeculectomy after 6 months (p = 0.003), 12 months (p = 0.01) and 18 months (p = 0.007) of observation. The filtering blebs after phacoemulsification in the study group were characterized by a greater reduction, compared to those in the control group. Cox regression survival success was 75% (SE = 5.9; 95% CI: 63.4 – 86.6), 75% (SE = 5.9; 95% CI: 63.4 – 86.6), 71% (SE = 5.4; 95% CI: 60.4 – 81.6) in study group and 92% (SE = 1.8; 95% CI: 91.5 – 98.5), 92% (SE = 1.9; 95% CI: 88.3 – 95.7), 91% (SE = 2.0; 95% CI: 87.1 – 94.9) in control group after 6, 12 and 18 months, respectively.

**Conclusions:**

Phacoemulsification causes a significant elevation of IOP in the eyes after previous successful trabeculectomy and deterioration of filtering bleb morphology.

## Background

Most authors agree that trabeculectomy remains the most efficient method of lowering the IOP [1-3], especially when augmented by the use of mitomycin C [4-6] or 5-fluorouracil (5-FU) [7,8]. The procedure is applied for the treatment of glaucoma which is refractory to pharmacological treatment, usually without significant lens opacity. Although the surgical technique of trabeculectomy has been improved for years, it is still associated with a relatively high risk of various complications [9-11].

Cataract, which is the most frequent late complication of trabeculectomy, constitutes an important problem concerning, according to different sources, from several to several dozen per cent of patients after a filtering surgery [12,13]. It has been shown that the filtration procedure itself may speed up the formation of lens opacity, for example in eyes with postoperative hypotony, shallow anterior chamber, or intense inflammation [14-17]. Clinically, it seems that phacoemulsification could have detrimental effect on the functioning filtering bleb, despite refinement of the surgical technique (small incision, gentle handling of the eyeball). However, opinions on this matter expressed over the last two decades are equivocal. The lack of consensus as to the effect of phacoemulsification following a successful trabeculectomy on the long-term IOP control and the appearance of filtering blebs made us undertake the present study.

Therefore, the objective of the retrospective study was to analyze in depth the effect of phacoemulsification with posterior chamber lens implantation on morphology and function of filtering blebs in patients after successful trabeculectomy.

## Methods

The study protocol approved by local ethical committee (Medical University in Lublin) adhered to the tenets of the Declaration of Helsinki. The retrospective study included 122 eyes of patients treated for primary open angle glaucoma in the Department of Ophthalmology of the Medical University in Lublin in the years 2004–2010, 50 eyes in which, after a successful trabeculectomy with 5-FU, temporal clear cornea phacoemulsification with posterior chamber lens implantation was performed (study group), and 72 eyes, in which a successful trabeculectomy was only performed (control group). The mean age of patients in the study group was 70.7 ± 7.0 years and 70.8 ± 6.3 years in the control group (the t-Student test). The study population consisted of 78 women and 44 men, of which 38 women and 12 men belonged to the study group, and 40 women and 32 men were in the control group. The mean interval between cataract surgery and antiglaucoma procedure was 19.9 ± 12.7 months (Table 1). The study included patients with open-angle glaucoma, in whom a successful trabeculectomy with antimetabolites (5FU) was carried out, i.e. in whom IOP measured after the antiglaucoma surgery was below 17 mmHg without antiglaucoma medications. Patients with angle-closure glaucoma, secondary glaucoma, those in whom perioperative complications occurred, or in whom antiglaucoma medications were ordered after the procedure were excluded from the study. The IOP was measured with an aplanation tonometer. We examined IOP and the appearance of filtering blebs before the procedure, 24 hours, 10 days, 1, 6, 12 and 18 months after phacoemulsification, and in patients who only underwent trabeculectomy 6, 12 and 18 months after study commencement.

**Table 1:** Description of the study population

**Parameter**		**Patients after Trab only**	**Patients after Trab then Phaco + PCIOL**	**Statistical**	
				**Analysis**	
**Number of eyes**		72	50	p = 0.05	
**Number of eyes with primary open-angle glaucoma**		72	50	p = 0.05	
**Gender**	**Women**	32.8%	31.2%	p = 0.02	
	**Men**	26.2%	9.8%		
**Mean age**	**Women**	71.8 ± 6.3 years	71.1 ± 8.3 years	p = 0.7	
	**Men**	69.8 ± 7.4 years	70.3 ± 5.7 years	p = 0.8	
**Time interval from trabeculectomy to first measurement of intraocular pressure included in the study**		19.2 ± 11.6 months	19.9 ± 12.7 months	p = 0.9	
**IOP at the beginning of the study**		11.0 ± 3.5 mmHg	11.6 ± 3.8 mmHg	p = 0.3	
**VF parameters**	**MD (dB)**	−19.6 ± 7.9	−19.3 ± 8.2	p = 0.9	
	**PSD (dB)**	7.9 ± 3.3	8.4 ± 2.6	p = 0.6	

Regarded as statistically significant if p < 0,05, (the chi-squared test, the t-Student test, the U Mann–Whitney test).

### Surgical technique

#### Phacoemulsification

Each phacoemulsification procedure was performed by an experienced surgeon and the technique used was the same throughout the study. All procedures were carried out in periocular or topical anaesthesia. First a clear-cornea temporal incision 2.6 mm long was made. After capsulorhexis and hydrodissection the nucleus of the lens was removed by means of phacoemulsification (Infiniti, Alcon, Fort Worth, USA), corneal remnants were washed out and aspirated with bimanual cannulas. Next, a posterior chamber hydrophobic foldable lens (Alcon AcrySof) was implanted, without viscoelastic, on BSS infusion only. The lens was implanted into the capsular bag. Surgery was concluded with closing the wound by hydratation, usually no sutures were needed. Occasionally, if needed, after synechiolysis, PMMA iris Morcher ring was applied to stretch the pupil.

### Statistics

The normality of variables distribution was evaluated by means of the Kolmogorov-Smirnov test. In order to assess differences between the analysed parameters Wilcoxon’s pair sequence test, sign test, Friedman test, U Mann–Whitney test, chi-squared test and t-Student test were all used. The Spearman’s correlation test were used to examine relationships between two parameters. The following correlation coefficient ranges were adopted: r = 0 (the variables are not correlated), 0 < r < 0.3 (small correlation), 0.3 < r < 0.5 (medium correlation), 0.5 < r < 0.7 (strong correlation), 0.7 < r < 0.99 (very strong correlation), and r = 1 (complete correlation). Cox regression was performed to evaluate the effect of cataract surgery on the trabeculectomy failure. For the purposes on study, we used criteria to define surgical success and surgical failure. The surgical success of the trabeculectomy was expressed as IOP < 17 mmHg and the surgical failure was defined as IOP ≥ 17 mmHg. A 5% concluding error was assumed and, consequently, the level of significance p < 0.05 indicated statistically significant differences or correlations. Statistical analyses were conducted by means of STATISTICA v. 8.0 (StatSoft, Poland).

## Results

The study group and the control group did not differ significantly as to the age of the study patients and IOP at the beginning of the study. To avoid bias, only subjects suffering from primary open angle glaucoma were included into the study The control group was constructed in such a way that the mean time after trabeculectomy was the same as in the control group (the chi-squared test; the t-Student test; the U Mann Whitney test) (Table 1).

In the study group the mean IOP in the period preceding phacoemulsification was 11.6 ± 3.8 mmHg. However, the mean IOP 24 hours, 10 days, 1, 6, 12 and 18 months after phacoemulsification were 14.4 ± 3.9 mmHg, 13.8 ± 3.4 mmHg, 13.5 ± 3.4 mmHg, 13.3 ± 3.5 mmHg, 13.1 ± 2.8 mmHg, and 13.2 ± 2.9 mmHg, respectively. The differences between mean IOP before phacoemulsification and mean intraocular pressure 24 hours (Z = 3.6; p = 0.0003), 10 days (Z = 3.7; p = 0.0002), 1 months (Z = 2.5; p = 0.01), 6 months (Z = 2.7; p = 0.007), 12 months (Z = 2.9; p = 0.003), 18 months (Z = 3.1; p = 0.002) after phacoemulsification were statistically significant. Mean IOP between consecutive measurements were also statistically analyzed, and a statistically significant difference was only revealed between the mean IOP before surgery and that 24 hours after surgery (Z = 3.6; p = 0.0003) (the Wilcoxon’s pair sequence test).

**Figure 1:**
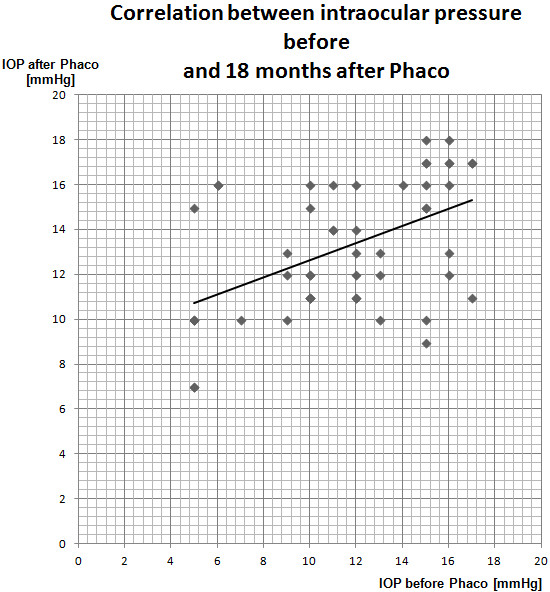
Correlation between IOP before and 18 months after phacoemulsification in the group of patients who previously underwent trabeculectomy (the Spearman’s correlation test).

In the study group (p = 0.0002; r = 0.5) and in the control group (p = 0,00009; r = 0.6) there was also a statistically significant strong positive correlation between IOP before phacoemulsification and IOP 18 months after the procedure. This means that the higher intraocular pressure before cataract surgery, the higher intraocular pressure was observed 18 months after the procedure. However, in the case of the control group the correlation was strong and in the study group it was medium (the Spearman’s correlation test) (Figure 1).

In the control group the mean IOP at the beginning of the study was 11.0 ± 3.5 mmHg, whereas 6, 12 and 18 months of study commencement it was 11.4 ± 3.4 mmHg, 11.4 ± 3.5 mmHg, and 11.5 ± 3.4 mmHg, respectively. In patients who only underwent trabeculectomy no statistically significant differences were observed between mean IOP at the beginning of the study and that obtained after 6 months (Z = 1.5; p = 0.1), 12 months (Z = 1.9; p = 0.06) and 18 months (Z = 1.9; p = 0.05) of observation. Similarly, the differences between mean IOP at consecutive stages of the study were not statistically significant (p > 0.05) (The Wilcoxon’s pair sequence test).

**Figure 2:**
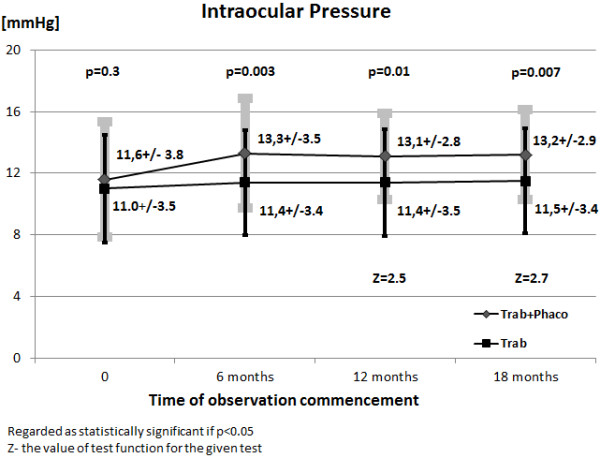
A comparison of mean intraocular pressures in the study group and the control group (the U Mann–Whitney test).

**Figure 3:**
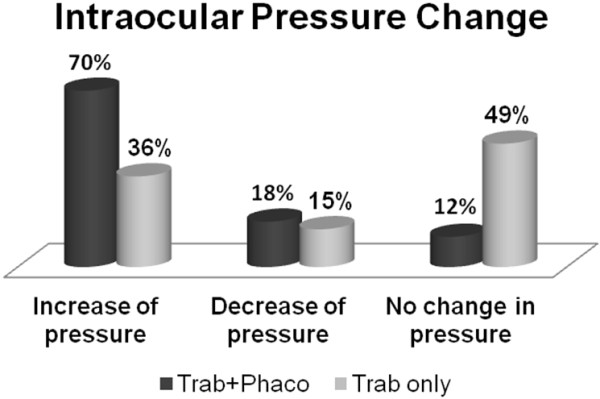
Percentage of subjects from study group and control group with mean intraocular pressure change, obtained 18 months of observation beginning against the initial result.

A comparison was also made between mean IOP in the study group and in the control group at particular study stages (6, 12 and 18 months). In the group of patients subjected to both trabeculectomy and phacoemulsification the mean IOP were significantly higher than in the group of patients who only underwent trabeculectomy after 6 months, 12 months and 18 months of observation. The difference between initial IOP in both groups was not statistically significant. (the U Mann–Whitney test) (Figure 2) It was demonstrated that in 70% patients after both trabeculectomy and phacoemulsification an increase in IOP occurred within 18 months of observation, whereas a similar increase occurred in only 36% patients of those who were subjected to trabeculectomy alone (Figure 3).

**Figure 4:**
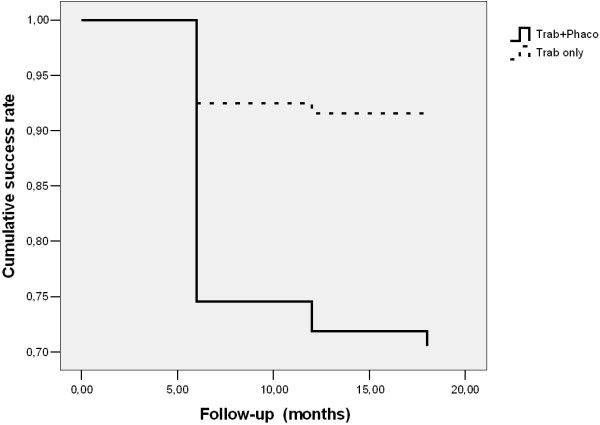
Cox regression for surgical success of trabeculectomy in the study group.

Cox regression was 75% (SE = 5.9; 95% CI: 63.4 – 86.6), 75% (SE = 5.9; 95% CI: 63.4 – 86.6), 71% (SE = 5.4; 95% CI: 60.4 – 81.6) in the study group and 92% (SE = 1.8; 95% CI: 91.5 – 98.5), 92% (SE = 1.9; 95% CI: 88.3 – 95.7), 91% (SE = 2.0; 95% CI: 87.1 – 94.9) in the control group after 6,12 and 18 months, respectively. In the control group it was revealed statistically significant higher probability of surgical successes in comparison to study group (Wald = 7.5; p = 0.006; Exp(B) = 3.7). The average time of failure occurring 14.6 months: M = 14.6 (SE = 0.8; 95% Cl: 13.1-16.2) in the study group and 17.2 months: M = 17.2 (SE = 0.3; 95% Cl: 16.5-17.8) in the control group. (the Cox regresssion) (Figure 4) Additionally we performed Cox regression for IOP 24 hours, 14 days and 1 month after cataract surgery in the study group. This analysis showed rate success 70% (SE = 5.4; 95% CI: 59.4 – 80.6), 54% (SE = 6.1; 95% CI: 42.0 – 66.0), 42% (SE = 6.1; 95% CI: 30.0 – 54.0), respectively 24 hours, 14 days and 1 month after phacoemulsification. The average time of failure occuring 7.77 months: M = 7.77 (SE = 1.2; 95% CI: 5.4 – 10.2) (the Cox regresssion).

The statistical analysis revealed that in the course of consecutive examinations there was a change in bleb surfaces (chi^2^(6) = 33.9; p < 0.001) and elevation (chi^2^(6) = 38.2; p < 0.001) in the study group (the Friedman test). In successive examinations filtering blebs in those who had undergone cataract surgery showed a tendency towards a reduction of size in time. There was a statistically significant difference in the study group between the surface and elevation of the blebs before cataract surgery and those from successive follow-up examinations (the sign test) (Table 2). On analyzing the consecutive examinations we noted significantly larger bleb surfaces 24 hours compared with 10 days (Z = 1.9; p = 0.05) after cataract surgery, as well as significantly larger bleb surfaces in a follow-up examination 10 days compared with 1 month after this procedure (Z = 2.2; p = 0.02). In the study group there was also a statistically significant lowering of the blebs 24 hours after the cataract operation with reference to those before the procedure (Z = 2.3; p = 0.02) (the sign test).

**Table 2:** Morphology of filtering blebs in the study group

**Time after phaco**			**Before Phaco**	**24 hours**	**10 days**	**1 month**	**6 months**	**12 months**	**18 months**
**Bleb Morphology**	**Surface**	Small	24%	24%	32%	38%	40%	40%	40%
		Medium	36%	58%	60%	58%	54%	54%	54%
		Large	20%	18%	8%	4%	6%	6%	6%
		Statistical analysis		Z = 0.3	Z = 1.6	**Z = 2.5**	**Z = 2.6**	**Z = 2.6**	**Z = 2.6**
				p = 0.8	p = 0.09	**p = 0.01**	**p = 0.009**	**p = 0.009**	**p = 0.009**
		(Before vs After Phaco)							

	**Elevation**	Elevated	96%	82%	76%	70%	66%	66%	66%
		Flat	4%	18%	24%	30%	34%	34%	34%
		Statistical analysis		**Z = 2.3**	**Z = 2.9**	**Z = 3.4**	**Z = 3.6**	**Z = 3.6**	**Z = 3.6**
		(Before vs After Phaco)							
				**p = 0.02**	**p = 0.004**	**p = 0.001**	**p < 0.001**	**p < 0.001**	**p < 0.001**

Regarded as statistically significant if p < 0,05, (the sign test).

Z – the value of test function for the given test.

No statistically significant changes within bleb surfaces (chi^2^(3) = 1.9; p = 0.6) or elevations (ch^2^(3) = 1.2; p = 0.7) were disclosed in the control group in the study period (the Friedman test). In that group the surfaces and elevations of the blebs in consecutive examinations 6, 12 and 18 months after the procedure in relation one to another and the blebs at the time of study commencement did not reveal statistically significant differences (p > 0.05). (the sign test) (Table 3).

**Table 3:** Morphology of filtering blebs in the control group

**Time after study commencement**			**Study**	**6**	**12**	**18**
			**Commencement**	**months**	**months**	**months**
**Bleb morphology**	**Surface**	Small	26.4%	26.4%	26.4%	27.8%
		Medium	45.8%	47.2%	47.2%	45.8%
		Large	27.8%	26.4%	26.4%	26.4%
		Statistical analysis		Z = 0.4	Z = 0.3	Z = 0.6
		(Commencement vs Consecutive Examinations)				
				p = 0.7	p = 0.7	p = 0.6
	
	**Elevation**	Elevated	86.1%	86.1%	84.7%	84.7%
		Flat	13.9%	13.9%	15.3%	15.3%
		Statistical analysis		Z = 0.0	Z = 0.6	Z = 0.6
		(Commencement vs Consecutive Examinations)		p > 0.9	p = 0.6	p = 0.6

Regarded as statistically significant if p < 0,05, (the sign test).

Z – the value of test function for the given test.

The filtering blebs after phacoemulsification in the study group were characterized by a greater reduction, compared to those in the control group at respective stages. No statistically significant differences were found within filtering bleb morphology at the beginning of the study in the control group and the blebs in the study group before cataract surgery (the U Mann–Whitney test) (Table 4).

**Table 4:** Comparison of bleb morphology in the study group against the control group

**Time after observation**			**Observation**		**6**		**12**		**18**	
**Commencement**			**Commencement**		**months**		**months**		**months**	
**Group**			**Trab then Phaco**	**Trab Only**	**Trab then Phaco**	**Trab Only**	**Trab then Phaco**	**Trab Only**	**Trab then Phaco**	**Trab Only**
**Bleb morphology**	**Surface**	Small	24%	26.4%	40%	26.4%	40%	26.4%	40%	27.8%
		Medium	36%	45.8%	54%	47.2%	54%	47.2%	54%	45.8%
		Large	20%	27.8%	6%	26.4%	6%	26.4%	6%	26.4%
		Statistical analysis	Z = 0.4		**Z = 2.6**		**Z = 2.6**		**Z = 2.4**	
			p = 0.7		**p = 0.01**		**p = 0.01**		**p = 0.01**	
	**Elevation**	Elevated	96%	86.1%	66%	86.1%	66%	84.7%	66%	84.7%
		Flat	4%	13.9%	34%	13.9%	34%	15.3%	34%	15.3%
		Statistical analysis	Z = 1.8		**Z = 2.6**		**Z = 2.4**		**Z = 2.4**	
			p = 0.07		**p = 0.009**		**p = 0.02**		**p = 0.02**	

Regarded as statistically significant if p < 0,05, (the U Mann–Whitney test).

Z – the value of test function for the given test.

No statistically significant correlation in the study group between the time interval from trabeculectomy to phacoemulsification and intraocular pressure (p = 0.3; r = −0.2), surface (p = 0.8; r = −0.05), or elevation (p = 0.8; r = −0.04) of filtering blebs 18 months after cataract surgery (the Spearman’s correlation test).

## Discussion

Despite the numerous studies on the effect of cataract surgery on the functioning of filtering fistula and filtering bleb, opinions on the subject still vary. Our study revealed a statistically significant rise in IOP 6, 12 and 18 months after temporal clear cornea phacoemulsification in the study group, as different from the control group. Similar results were obtained by Casson and co-workers [18], who analysed IOP in patients after previous trabeculectomy subjected to phacoemulsification with incision in the upper part of the clear cornea, and in the control group who underwent trabeculectomy only. Mean IOP 12 months after cataract operation was 15.6 ± 3.5 mmHg in the study group and it was significantly higher than mean IOP in the control group, which was 13.4 ± 2.5 mmHg. Measurements of IOP performed 24 months after cataract operation were: 15.3 ± 3.1 mmHg and 14.3 ± 3.2 mmHg, respectively, but the difference was not statistically significant. Rebolleda and Muñoz-Negrete [19] reported that an increase in intraocular pressure was significant during all measurements that they performed during 24 months observation. The percentage of patients using antiglaucoma medications also went up systematically. Inal and co-workers [20] observed a deterioration in IOP control and a growing frequency and amount of necessary antiglaucoma medications both in the study group and in the control group. In our study all participants did not receive any antiglaucoma medications what better reflected IOP changes after phacoemulsification. Other authors also noted similar results [21-23]. All trabeculectomies included in our study were augmented with antimetabolite agent 5-FU and therefore it would be more appropriate to compare our results to those of Swamynathan and co-workers [24]. In this study the effect of temporal corneal phacoemulsification on intraocular pressure in eyes after primary trabeculectomy with intraoperative 5-FU or mitomycin C was evaluated. The analysis revealed that the mean of all IOP measurements beyond 3 months after phacoemulsification for each subject was significantly higher than the pre-phacoemulsification IOP. Also post-phacoemulsification IOP was significantly higher than the corresponding IOP in the time-matched control group. Similar results were observed in our study, although unfavourable IOP increase in relation to IOP before phacoemulsification was noted in the first month after this procedure. Considering a rise in IOP after cataract surgery in the eyes previously subjected to trabeculectomy, one must bear in mind the hypotensive effect of phacoemulsification itself on one hand and normal course of IOP after filtering surgery on the other hand. There are numerous reports in the literature which confirm hypotensive effect of phacoemulsification. Jampel and co-workers [25] estimated the hypotensive effect of phacoemulsification at 1–3 mmHg. Similar opinions came from other authors who confirmed a drop in IOP after phacoemulsification by a few millimeters Hg followed up for 12 months [26-28]. But data showed in the Advanced Glaucoma Intervention Study (AGIS) revealed slight increase of IOP over time after trabeculectomy [14]. An average increase of IOP during 72 months of observation was equal to 0.33 mmHg in the group with IOP < 14 mmHg, 1.11 mmHg in the group with IOP 14–17 mmHg and 2.6 mmHg in the group with IOP >17.5 mmHg. Therefore, the influence of phacoemulsification on IOP in eyes after previous trabeculectomy seems to be a result of several processes.

Our study showed that phacoemulsification may be responsible for decrease of hypotensive effect of trabeculectomy in long-term observation. The estimated Cox regression success rates in control groups were 92%, 92%, and 91% at 6 months, 12 months, and 18 months, respectively. In the study group success rates were 75%, 75%, and 71% at 6 months, 12 months, and 18 months, respectively. In that case the decrease of success rate of trabeculectomy occurred during 6 months following phacoemulsification, later it was stabilized. However, other authors reported that success rates were decreased later than after 6 months. Rebolleda and Muñoz-Negrete [19] reported success rates at the level of 87.8%, 83.6%, 68.2%, 63.6%, and 55.7% at 3 months, 6 months, 12 months, 18 month, and 24 months after phacoemulsification in eyes which underwent previous phacoemulsification. Similar success rates were demonstrated by Crichton and Kirker [21] – 86.9%, 81.9%, 75.6%, and 72.5% after 6 month, 12 months, 18 months, and 24 months, respectively. Above mentioned results suggest that phacoemulsification may affect trabeculectomy effectiveness during long period of time. The most important limitation of above mentioned results is the arbitrary definition of postoperative success that was used in different studies that makes comparisons very difficult.

The other aspect of our study was an analysis of the effect of cataract surgery on the morphology of filtering blebs. The appearance of filtering blebs is an indirect reflection of the function of fistula formed during the operation, although there is no direct relationship between its morphology and the level of intraocular pressure. Surgical incision in the conjunctiva is suggested as a critical factor for bleb formation after trabeculectomy with fornix-based conjunctival flaps lead to diffuse blebs extending into the conjunctival fornix contrary to limbal-based conjunctival flaps developing cystic blebs demarcated in the region between the corneal limbus and the incision in the fornix [29]. However, Study of Keiichiro and co-workers [30] showed no significant association between IOP and bleb formation. Postoperative IOP is thought to be derivative from many various factors such as postoperative inflammation, aqueous humor production from the ciliary body, and the outflow through the fistula created by trabeculectomy as well as through trabeculum and uveoscleral pathway.

Perhaps also other factors influence the drainage process in eyes after filtration surgery. In a normal eye there is no connection between the conjunctival lymphatics and the aqueous humour drainage system. After glaucoma filtering surgery aqueous humour is directed to the subconjunctival space. Not only passive diffusion and trans-epithelium pathway are involved in drainage process. It is suggested that formation of drainage pathways particularly lymphatic vessels plays critical role in maintaining long term drainage pathway. Additionally, glaucoma filtration surgery results in changes of aqueous humour properties because it can bypass the cleaning function of trabecular meshwork. This aqueous humour then enters the highly immune-reactive conjunctival tissue and can be responsible for induction of inflammatory response [31].

Our studies revealed reduction of filtering blebs size and elevation in eyes after cataract surgery corresponding to compromise of IOP control. In fact, unfavourable alterations in filtering bleb morphology following phacoemulsification, consistent with our observations, were already reported. Inal and co-workers [20] described a decrease in the surface and degree of elevation within the filtering blebs both in the control group subjected only to trabeculectomy and in the study group. The factor which differentiated both groups was the pace of change. In the control group the reduction of filtering blebs occurred slowly with time, and in the study group the changes were more abrupt in character, occurring shortly after phacoemulsification procedure. The explanation that the authors proposed was a reduction in the formation of aqueous humour, or its more intensive outflow via uveoscleral route, due to an inflammatory process induced by the phacoemulsification procedure. Others also observed a reduction of the surface of filtering blebs and their flattening after removal of cataract in the eyes previously subjected to trabeculectomy. The differences mainly concerned the percentage of patients with adverse changes. Rebolleda and Muñoz-Negrete [19] described the above-mentioned effect in 77.6% of patients, Wygnanski-Jaffe and co-workers [32] in more than 70% of patients, Yamagami and co-workers [33] in 65%, Chen and co-workers in 18% of patients [34]. In our study filtering bleb showed tendency to size limiting mainly during first 6 months, at most to 1 year after phacoemulsification. However, mechanisms responsible for worsening of filtering fistula function and a reduction of filtering blebs after cataract surgery are not fully explained. Phacoemulsification leads to an increase in the permeability of blood–aqueous humour barrier, thus inducing an inflammatory process and contributing to fibrosis. Żarnowski and co-workers [35] used laser flare-meter to examine the intensity of inflammatory process following procedures in the anterior segment of the eye. They noted a marked elevation of tyndalometry values after a triple procedure, slightly smaller as a consequence of trabeculectomy, and the lowest after phacoemulsification.

The deposition of collagen fibers was confirmed by histological examinations of conjunctival preparations taken from patients during reoperations carried out at different times after cataract removal. Additionally, it was shown a statistically significant correlation between the level of IOP before cataract operation and the degree of filtering bleb reduction after the operation. Intraocular pressure before the removal of the opaque lens which was higher than 10 mmHg was associated with a significantly higher risk of filtering bleb dysfunction in the postoperative period [19]. Other authors enumerated factors which are responsible for a remarkably worse functioning of filtering fistula and hinder intraocular pressure control following cataract procedure after previous filtering surgery operations in the eyes. They included: age below 50 years, preoperative IOP higher than 10 mmHg, iris manipulations during the procedure (synechiolysis, sphincterotomy, use of retractors, iridectomy, suturing of the iris), and an increase in intraocular pressure above 25 mmHg shortly after the operation. It is also believed that time period between trabeculectomy and cataract surgery is a very important factor for the maintenance of the function of filtering fistula [34]. Chen and co-workers [34] showed that time interval of 6 months or shorter is associated with a significantly higher risk of failure following cataract surgery. Other author suggested that a proper formation of the filtering blebs, which would make it more resistant to harmful factors, involved at least 12 months period [36].

Taken together, our study showed a remarkable deterioration in the functioning of filtering fistula, bringing about a rise in intraocular pressure and adverse alterations in the morphology of filtering blebs, as a consequence of phacoemulsification of cataract in eyes formerly subjected to trabeculectomy. The most significant influence of phacoemulsification to adverse changes of filtering fistula was observed during first 6 months after cataract surgery.

Our study has some limitations such as retrospective nature, performing surgical procedures by more than one surgeon and evaluation of filtering bleb morphology based on only two parameters – size and height. Despite these limitations our data can be treated as another one voice in discussion referring to the problem of filtering fistula function after phacoemulsification. This allows us to say that, in spite of progress in surgical techniques that has happened recently, cataract operations pose a serious risk of worsening glaucoma control in the eyes after trabeculectomy, so that considering a patient for those procedures should be carefully considered in each individual case. An option to avoid this problem could be performing phacotrabeculectomy more often. Especially that some authors consider that the evolution and refinement of small incision technique has made combined surgery an attractive option and has meant that a large proportion of patients with coincident cataract and primary open-angle glaucoma will have phacotrabeculectomy as the procedure of choice. They consider that most evidence indicates that a combined procedure using small incision phacoemulsification coupled with trabeculectomy is safe and that an IOP reduction of 5.0 to 8.0 mmHg can be obtained [37]. However, target pressure around low teens might be difficult to achieve with this technique even with MMC. Nevertheless, chosen surgical strategy depends on the target pressure, the amount of glaucomatous damage, and the grade of visual disturbance caused by cataract [37,38]. Thus, bearing in mind advantages and drawbacks of all options the decision should be individualized.

## Conclusions

Phacoemulsification causes a significant elevation of IOP in the eyes after previous successful trabeculectomy as well as deterioration of filtering bleb morphology. Phacoemulsification after trabeculectomy increases the risk of trabeculectomy failure.

## Competing interests

The authors declare that they have no competing interests.

## Authors’ contribution

MS-P: design, data acquisition, analysis and interpretation of data, drafting the manuscript. MK: data acquisition, analysis and interpretation of data, drafting the manuscript. TŻ: design, coordination, revising the manuscript critically. All authors read and approved the final manuscript.

## Pre-publication history

The pre-publication history for this paper can be accessed here:

http://www.biomedcentral.com/1471-2415/13/17/prepub
